# Mixture for the Teeth

**Published:** 1858-01

**Authors:** 


The following is an excellent deutrifice to neutralize the products of decomposition and stimulate the gums, while it imparts an agreeable aroma to the breath:

| | | |
|---|---|---|
| ℞ | Cochinelle, | 3 grains. |
| | Sub. Carb. Potass, | 5 " |
| | Distilled water, a few drops. | |
| | Essence of Peppermint, | 4 drachms. |
| | Alcohol, | 1 quart. |

Wet the pulverized cochinelle and sub. carb. potass with the water, enough to thoroughly moisten together, and then put them into a bottle containing the alcohol and mint previously mixed. By using this mixture, first washing the mouth well with water, there need be no troublesome accumulation of tartar or inflammation of the gums, which so frequently cause the teeth to decay and fall out.—*L'Art. Dentaire.*

